# Oral health promotion in early childhood: age of joining preventive program and behavioral aspects

**DOI:** 10.1590/S1679-45082014AO2895

**Published:** 2014

**Authors:** Letícia Vargas Freire Martins Lemos, Silvio Issáo Myaki, Luiz Reynaldo de Figueiredo Walter, Angela Cristina Cilense Zuanon

**Affiliations:** 1 Universidade Estadual Paulista “Júlio de Mesquita Filho”, São José dos Campos, SP, Brazil; 2 Universidade Estadual de Londrina, Londrina, PR, Brazil; 3 Universidade Estadual Paulista “Júlio de Mesquita Filho”, Araraquara, SP, Brazil

**Keywords:** Dental caries/epidemiology, Dental care for children, Behavior, Health education, dental, Child

## Abstract

**Objective:**

To analyze the interference of age in the entrance into a public dental care program for infants as well as family behavioral aspects about tooth decay experience in children 0 to 4 years old.

**Methods:**

Cross-sectional study involving 465 children who were divided into 3 groups: infants whose mothers joined the program during pregnancy (n=50); infants enrolled in the program during the first year of life (n=230); and infants enrolled in the program between 13 and 18 months old (n=185). The χ^2^ and Kruskal-Walis tests (95% confidence interval) were used to assess the relationship among variables.

**Results:**

There was an association between the age of entrance in the programs and dental caries (p<0.001). A lower prevalence was seen in infants whose mothers joined the program during pregnancy, and among those infants enrolled in the program during the first year of life. The same low prevalence occurred in relation to mothers' commitment to attend follow-up visits with their infants, cariogenic diet, nighttime oral care, duration of night feeding and parents' educational level (p<0.001). Unfavorable socioeconomic conditions (p>0.05) and daily oral care (p=0.214) were common variables in the groups with 99% of occurrence. Commitment to attend follow-up visits, nighttime oral care and parents' educational level (p>0.05) were considered protective factors for dental caries. Cariogenic diet and night feeding were determinant factors to the appearance of dental caries.

**Conclusion:**

To promote children oral health it is essential to enroll children in oral health programs and adopt healthy habits as early as possible, besides the adherence of the child to their parents' advice.

## RESUMO

**Objetivo:**

Analisar a interferência da idade de ingresso em programas públicos de Odontologia para crianças e dos aspectos comportamentais familiares sobre a experiência com cárie dentária em crianças de zero a 48 meses de idade.

**Métodos:**

Estudo transversal com 465 crianças divididas em 3 grupos: crianças cujas mães ingressaram no programa quando gestantes (n=50); crianças que ingressaram no programa durante o primeiro ano de vida (n=230); e crianças que ingressaram no programa entre 13 e 18 meses de idade (n=185). Os testes χ^2^ e Kruskal-Walis (intervalo de confiança de 95%) foram utilizados para avaliar a relação das variáveis.

**Resultados:**

Encontrou-se associação entre a idade do ingresso nos programas e cárie dentária (p<0,001), sendo menor a prevalência para crianças cuja as mães ingressaram no programa durante as gestação e para aqueles que ingressaram no programa durante o primeiro ano de vida, o mesmo ocorreu em relação a assiduidade as consultas de retorno, dieta cariogênica, higiene oral noturna, duração de aleitamento noturno e escolaridade materna/responsável (p<0,001). Desfavorável nível socioeconômico (p>0,05) e higiene oral diária (p=0,214) foram variáveis comuns nos grupos com 99% de ocorrência. Assiduidade, higiene oral noturna e escolaridade materna (p<0,05) foram consideradas fatores de proteção à cárie dentária. Dieta cariogênica e aleitamento noturno foram consideradas determinantes para o aparecimento da cárie dentária.

**Conclusão:**

Para promover saúde oral infantil é essencial o ingresso ao programa e adoção de hábitos saudáveis de maneira precoce, além da adesão às orientações por seus responsáveis.

## INTRODUCTION

Dental caries is the major problem in oral health because of its prevalence and severity.^(1,2)^ Tooth decay is presented as a common chronic disease in childhood and a great public health problem worldwide. To date, a single epidemiological survey carried out by the *Brazilian Health Ministry* ^(3)^ in oral health area including children aged 18 to 36 months old showed that 27% of them had at least one decayed tooth in deciduous teeth. Despite the currently decline of this disease^(4–6)^ in Brazil and around the world, early childhood caries (ECC) can develop particular characteristics of such gravidity that may interfere in the development and growth of children. So, caries will be considered as a health problem in childhood affecting society with all implications it could bring, and not only as a specific dental problem.^(6)^ This disease could be prevented, controlled and reversed. For this reason, the team work of physicians, and especially those professionals dealing with children in the first year of life, is important to enable the earliest referral of children to the dentist.

The program “Dentistry for Infants” began in Brazil in 1984 at *Universidade Estadual de Londrina* (UEL). Since that time adequate equipment and care system were developed for health promotion and prevention of dental caries named “Baby Clinic”.

In 1996,^(7)^ the adoption of the same model of “Dentistry for Infants”, a public health preventive program in basic health unit (UBS, acronym in Portuguese) was started at the municipality of Jacarei. In that program, the assistance starts with an educational action (lecture) that aimed to encourage families to self-care. Consultations were conducted periodically with intervals from 1 to 3 months until the child turns 4 years old according to assessment on risk of dental caries development. In 2000, the *Faculdade de Odontologia de São José dos Campos* of the *Universidade Estadual Paulista “Júlio de Mesquita Filho”* (UNESP) launched a project named “Living without tooth decay” that provided assistance to pregnant women by offering educational lectures. Infants were followed from 0 to 4 years old.

The practice of “Dentistry for Infants” has as it first source of attention the parents/responsible, and it is revealed as co-participative and solidary dentistry^(7)^ in which parents' behavior will affect directly the health of their children whom are starting to form habits. The special stimulus for self-care focused on health is stressed, being this stimulus essential to the child oral health promotion.^(8)^

A great obstacle to the success of preventive programs has been the lack of commitment of families to instruct their children in oral hygiene,^(9–11)^ besides the early adoption of inadequate habits that cause oral diseases. Behavioral habits such as commitment to attend follow-up visits,^(12)^ presence of non-cariogenic diet, oral hygiene and duration of night feeding could interfere in maintenance of oral health of a child in early childhood. For example, the assiduity in follow-up visits is the responsibility of the infants families who are participating in preventive programs that show the adherence of families to given information and level of valorization to oral health. Therefore, it justifies the study of existing relations between factors reported here and behavior of parents/responsible that show consciousness and apply preventive daily measures to children at home.

## OBJECTIVE

To analyze the interference of age entrance of children in dental care programs and the behavioral aspects of the family related to dental caries experience.

## METHODS

Cross-sectional analytic study conducted between 2011 and 2012 at units of public health network in the city of Jacareí. We analyzed two preventive programs of “Dentistry for Infants” classified as group G1 and group G2. Another group with the same design of the *Faculdade de Odontologia de São José dos Campos da* UNESP was included and classified as group G0. Jacareí is a city of 211,214 inhabitants,^(13)^ whereas the population of the municipality of São José dos Campos (SP) is 629,921.^(14)^ All individuals included in the study from both cities live in urban areas and receive the same dosage of fluorine in the water (0.7ppmF).

The programs' protocols and target-audience were similar differing only on the age of enrollment of children in the programs.

Eligibility criteria for inclusion were children no older than 4 years old of every gender and race and who participated in preventive program for at least 3 months, and having at least central superior and inferior incisor teeth in the oral cavity. Exclusion criteria were presence of systemic disease or syndromes, and use of medicines that could cause stains or changes to tooth enamel.

The sample included 465 children (6 months to 4 years old) who met the eligibility criteria. Participants were divided into 3 groups: G0: infants whose mother joined the program during pregnancy (G0; n50); infants enrolled in the program in first year of life (G1; n=230); and infants enrolled in the program between 13 and 18 months old (G2; n=185). For data collection we used a visual-tactile clinical examination by a single calibrated operator and also a questionnaire applied to the children responsible is in order to analyze diet habits, morning and night oral hygiene, duration of nighttime feeding, mother/responsible's formal education level and socioeconomic condition.

This study was approved by the ethical and research committee of *Faculdade de Odontologia de Araraquara da* UNESP, protocol number 47/10-PH/CEP. All parents signed the consent form.

Variables analyzed in this study were age when enrolled in the program, dental caries (prevalence), assiduity, cardiogenic diet, daily oral hygiene, nighttime oral care, duration of nighttime feeding, mother/responsible's formal education level and socioeconomic condition.

We used χ^2^ and Kruskal-Wallis (software PASW 20.0) tests to compare groups. When a difference was found, we conducted non-parametric multiple comparisons (CI 95%). In addition, odds ratios (OR) were calculated with adjustment of logistic regression.

## RESULTS

Table 1 shows frequencies and variable percentages analyzed in relation to the total of each group. Daily oral hygiene and unfavorable socioeconomic level were not associated with groups because almost all individuals had the same characteristics. The variables dental caries, assiduity, nighttime oral care and mother/responsible's formation education level and socioeconomic condition were independent in relation to groups G0 and G1, but they had significant association compared to G2. Cariogenic diet was associated with the three groups. We observed that the proportion of children with dental caries increased significantly when group G0 to G2 were analyzed, which confirmed the association of dental caries prevalence to the age of enrollment in the programs (p<0.001). Analyzing tooth decay as disease, the groups G0, G1 and G2 presented prevalence of 16%, 12% and 31%, respectively.

**Table 1:** Frequency (percentage proportion on total of each group) of each classification of the variable in groups of infants whose mother was enrolled in the program during pregnancy (G0), infants enrolled in the program in the first day of life (G1) and infants enrolled in program between 13 and 18 months old (G2)

Variable		G0	G1	G2	p value*
Dental caries					
	No	42 (84)	202 (88)	127 (69)***	<0.001
	Yes	8 (16)	28 (12)	58 (31)	
Assiduity					
	No	13 (26)	69 (30)	93 (50)***	<0.001
	Yes	37 (74)	161 (70)	92 (50)	
Cariogenic caries					
	No	40 (80)	125 (54)**	49 (26)***	<0.001
	Yes	10 (20)	105 (46)	136 (74)	
Daily oral hygiene					
	No	0 (0)	3 (1)	0 (0)	0.214
	Yes	50 (100)	227 (99)	185 (100)	
Nighttime oral care					
	No	17 (34)	26 (11)	41(22)***	<0.001
	Yes	33 (66)	204 (89)	144 (78)	
Socieconomic condiction					
	Favorable	0 (0)	3 (1)	0 (0)	0.214
	Unfavorable	50 (100)	227 (99)	185 (100)	
Mother/responsible formal education level					
	Up to 8 years	37 (74)	194 (84)	169 (91)***	0.004
	More than 8 years	13 (26)	36 (16)	16 (9)	

* p value of χ^2^ test associating G0, G1 and G2;

** significant association between G0 and G1;

*** significant association between (G0+G1) and G2.

**Figure 1:**
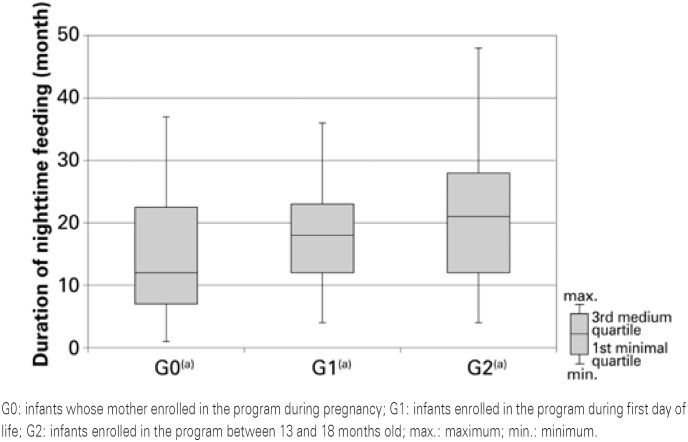
Graphic representation of descriptive statistics of age, duration of nighttime breastfeeding in months (Krsuskal-Wallis test; p<0,001). Groups identified with letters “a” are not significantly different

The duration of nighttime feeding in months seen in figure 1 emphasizes equivalences between group G0 and G1, but there was no difference in these groups when compared with G2.

The variables assiduity (OR=0.03), presence of nighttime oral hygiene (OR=0.04) and mother/responsible with more than 8 years of formal education (OR=0.23) constitute protective factors against dental caries. Cariogenic diet (OR=18.725) and nighttime feeding (OR=4.85) were considered as determinants for dental caries.

## DISCUSSION

To know the population's health profile contributes to improve the development of public policies for children.

Important studies^(4–7,10,11)^ have emphasized that the greater the number of aspects of children' routine assessed in a study together with their parents/responsible (because they certainly will reflect in life of the very young child) the higher will be the reliability of the context showed in the study. Therefore, this study showed that when the three previously mentioned preventive programs were analyzed, using similar protocols but with different ages of enrollment, it was possible to draw differences that could occur in oral health of those children.

Among the results found, there was an association between children's age of entrance in the programs and dental caries; although most children did not have dental caries, some of them had the disease. These data agree with the literature.^(15)^ Children whose mothers enrolled in the program during pregnancy (G0) had lower dental caries prevalence as well as those children enrolled in the first year of life (G1). This result was different for those children who started the program in the second year of life (G2). Analyzing the prevalence in G2 (31%) and comparing to those reported by the Brazilian Health Ministry^(3)^ of 27%, it is possible to assure that in relation to development of dental caries, these children are in similar situations. However, this resemblance should not occur from the disease is severity point of view because in these program children who developed dental caries, developed them in early stages (white stains) and, in several cases, they are reversible.

Freire et al.^(16)^ reported that children from 0 to 6 years old with unfavorable socioeconomic status had high prevalence of caries. In our study we also observed that in children who still live in such conditions caries prevalence could be low when they are precociously enrolled in a preventive program.

We know that inappropriate food consumption within the first years of life is associated with the appearance of several diseases, such as dental caries. The cariogenic diet, which showed association with the three groups, is determinant of the impact of dental caries.^(17)^ However, the participation in the program during gestation influenced the children's diet given that 80% of G0 did not follow a cariogenic diet, 46% of G1 followed a cariogenic diet and 74% of G2 had a cariogenic dietary habit. Associating these data with the increase of dental caries in the groups, we observed that data a parallel evolvement from G0 to G2. Therefore, based on this study findings, we could state that the enrollment in preventive programs as early as possible the better will be the protection against the development of dental caries even in children with unfavorable socioeconomic conditions.

In addition, we evaluated oral daily care (supervised toothbrushing) which did not present association with age of children enrollment in the programs because almost all participants of the three groups (99%) had a good dental care habit. This fact suggests the efficacy of preventive programs concerning adoption of new healthy habits. When more complex hygiene was analyzed^(17)^, *i.e.*, the nighttime oral care along with the dental care habit at night (oral hygiene after night feeding), the children of G1 had the highest number of individuals with dental care habit, being also the group with lowest prevalence of dental caries.

From the point of view of assiduity to follow-up visits we observed that infants from the G0 and G1, who received oral education before and during the first year of life, the percentage of assiduity found was higher than in G2.

We also observed the positive impact of the program on duration of night feeding, which was similar between G0 and G1 and both presented a difference in relation to G2, therefore, following again the variable profile of dental caries. This statement is supported by the literature^(6,7,15)^ that report that the most effective educational measures to prevent dental caries in early childhood are those directed to control night feeding. This habit is characterized by a pattern of high frequency, long period of feeding and inadequate time for feeding (before going to sleep or during sleeping).

Persson et al.^(18)^ stated that mothers/responsible's formal education level interferes with the feeding pattern of children feeding and, as a matter of fact, on their dental caries prevalence. In addition, cultural and social aspects that lead to early weaning require artificial feeding therefore leading to early introduction of sucrose in the child's diet.^(7)^ Other authors^(17,19,20)^ affirmed that the mother's figure has the highest influence in food education and nuclear family habit. Therefore, in this study we analyzed mothers/responsible's formal education level that was associated with age of enrolling in the program. The efficacy of the program that corresponded to G0 and G1 were seen by the results of this study.

Further studies on determinants of dental caries should be conduct to improve oral health promotion measures.

## CONCLUSION

To promote children's oral health is essential to enroll children in oral health program and adopt healthy habits as early as possible, besides the adherence of the child to parents' advice.
